# Differential effects of gram-positive and gram-negative bacterial products on morphine induced inhibition of phagocytosis

**DOI:** 10.1038/srep21094

**Published:** 2016-02-19

**Authors:** Ninkovic Jana, Anand Vidhu, Dutta Raini, Li Zhang, Anuj Saluja, Jingjing Meng, Koodie Lisa, Banerjee Santanu, Roy Sabita

**Affiliations:** 1 Department of Surgery, University of Minnesota, Minneapolis, MN 55455; 2 Department of Pharmacology, University of Minnesota, Minneapolis, MN 55455.

## Abstract

Opioid drug abusers have a greater susceptibility to gram positive (Gram (+)) bacterial infections. However, the mechanism underlying opioid modulation of Gram (+) versus Gram (−) bacterial clearance has not been investigated. In this study, we show that opioid treatment resulted in reduced phagocytosis of Gram (+), when compared to Gram (−) bacteria. We further established that LPS priming of chronic morphine treated macrophages leads to potentiated phagocytosis and killing of both Gram (+) and Gram (−) bacteria in a P-38 MAP kinase dependent signaling pathway. In contrast, LTA priming lead to inhibition of both phagocytosis and bacterial killing. This study demonstrates for the first time the differential effects of TLR4 and TLR2 agonists on morphine induced inhibition of phagocytosis. Our results suggest that the incidence and severity of secondary infections with Gram (+) bacteria would be higher in opioid abusers.

Opioid mediated suppression of immune system has been documented by a wealth of publications^1^. Deleterious effects of morphine-induced immune suppression have most frequently been observed in opioid users and abusers, where opioid use have been shown to increase susceptibility to infection, impaired phagocytosis and inhibition of bacterial clearance thereby leading to increased dissemination of bacteria and sepsis^2^,^3^. Epidemiologic studies provide data on increased prevalence of opportunistic, gram-positive bacterial infections, such as pneumonia, in opioid users and abusers. The incidence of pneumonia and tuberculosis is ten fold higher in intravenous drug users (IDUs) when compared to the normal population^4^. A recent study in IDU patients from Canada showed, that the two most common reasons, in 74% of the IDU patients admitted in emergency departments, over a three year period, was pneumonia and soft-tissue infections^5^.

The epidemiological studies on pulmonary infections among IDUs are supported by *in vivo* and *in vitro* data, where chronic morphine treatment increased susceptibility and mortality in murine model of *Streptococcus pneumonia* through inhibition of neutrophil migration to the site of the infection and through impairment of alveolar macrophage phagocytosis and decreased bacterial clearance^3^,^6^. Other investigators reported similar deleterious effects of chronic opioid use in animal models in response to *Klebsiella pneumoniae*, *Toxoplasma gondii*, *Salmonella typhimurium*, *Candida albicans*, *Staph aureus*, and *Listeria monocytogenes*^7^,^8^,^9^,^10^,^11^. However, gaps still exist in understanding the mechanisms leading to increased bacterial loads following morphine treatment. One of the ways by which morphine increases bacterial dissemination is by modulating macrophage function, specifically inhibiting phagocytosis and modulating Toll-like receptor (TLR) mediated cytokine release^12^,^13^. Morphine has been known to inhibit immune function by modulating inflammatory pathways mediated by TLRs such as inhibition of cytokine production as well as NFκB expression^7^,^13^,^14^,^15^. However we have previously showed that the effects of morphine are biphasic. The early response is inhibitory with morphine decreasing pro-inflammatory cytokine release; but long term treatment results in impaired endotoxin tolerance development leading to sustained cytokine secretion and persistent inflammation^16^.

TLR2 and TLR4 are the most extensively studied members of the TLR family which recognizes bacterial products from Gram-positive bacteria, such as lipoteichoic acid (LTA), and Gram-negative bacteria, such as lipopolysaccharide (LPS), respectively.

Even though the primary role of TLRs is to initiate inflammatory pathways, they have also been implicated to play a role in the modulation of macrophage phagocytosis. TLR activation is known to affect phagocytosis in three steps 1) by potentiating phagosome formation, 2) by affecting phagosome maturation and 3) by leading to transcriptional responses that affect genes transcription in all steps of phagocytosis^17^. Microarray analysis indicates that prolonged TLR3, TLR4 and TLR9 stimulation upregulates the FcγR receptor and scavenger receptors thereby resulting in increased phagocytosis^18^,^19^. Mechanistically it has been demonstrated that TLR-induced macrophage phagocytosis is mediated through a MyD88–IRAK4–p38-dependent pathway^17^.

However, no studies have examined how TLR activation during chronic morphine treatment modulates phagocytosis. Considering current literature and observations that toll-like receptors signaling may be interacting with opioid receptors to modulate macrophage phagocytosis^20^, the aim of this study is to examine the effects of activation of TLR2 and TLR4 by LTA and LPS respectively on morphine mediated inhibition of phagocytosis.

Our results demonstrate that chronic morphine treatment inhibits phagocytosis of gram-positive bacteria more than gram-negative bacteria , which could account for increased incidence and severity of gram positive infections in chronic opioid users and abusers. We further show that LPS priming increases macrophage phagocytosis whereas LTA inhibits it which is consistent with previous studies^21^,^22^,^23^,^24^. Our results show for the first time the differential effects of LPS and LTA priming on morphine induced inhibition of phagocytosis. We report that LPS priming increases phagocytosis in chronic morphine treated macrophages which is higher than the effects of LPS alone. We show that LPS induced effects are a consequence of increased TLR4 mRNA and protein expression with morphine treatment. In contrast, we do not observe any priming effects with LTA. We further show that LTA mediated inhibition was neither additive nor synergistic to opioid induced inhibition of macrophage phagocytosis, which is probably because of the ceiling effect reached by morphine treatment alone. Our results also indicate worse prognosis of secondary infections after a gram positive infection. There is a need of well-conducted clinical studies looking at the outcomes of abusers with aggressive management of gram-positive infections.

Elucidation of mechanisms by which LPS and LTA modulates bacterial phagocytosis and clearance in the context of opioids will add to our understanding and treatment of infections in chronic opiate users and abusers.

## Results

### Modulation of phagocytosis by chronic morphine treatment following LPS and LTA priming

Opioid use and abuse has been implicated in increased susceptibility to opportunistic infections, bacteremia and sepsis. Literature shows that morphine inhibits innate immune function, resulting in inhibition of clearance and increased dissemination of bacteria. However a gap in knowledge still exists in understanding if and how LPS and LTA priming, affects morphine induced inhibition of macrophage phagocytosis.

**Figure 1:**
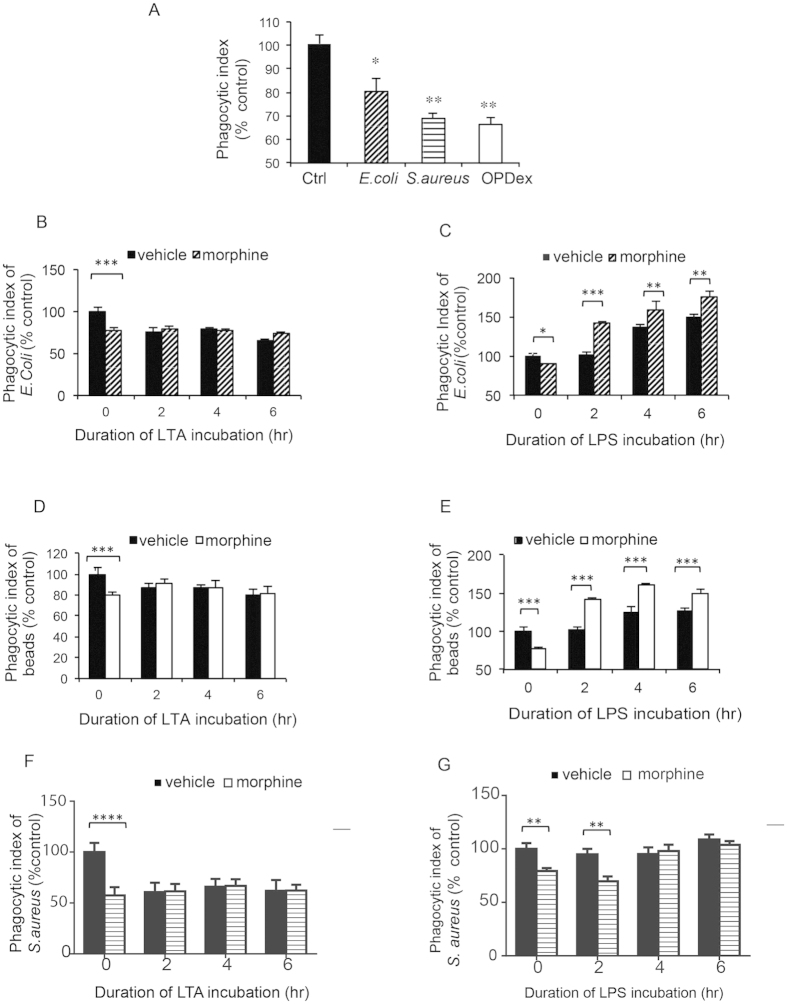
Differential effects of TLR4 (LPS) and TLR2 (LTA) ligands on phagocytosis of chronic morphine treated macrophages. (**A**) Fluorometric analysis of phagocytosis of HKO*E. coli (30 min),* HKO*S. aureus* (30 min) and OPDex bead (60 min) by J774 following chronic morphine treatment (1 μM overnight). Phagocytosis of opsonized HKO *E. coli* (**B,C**) HKO*E. coli* and (**D,E**) OPDex bead (**F,G**) HKO*S. aureus* by morphine/vehicle treated J774 macrophages following LTA (10 μg/ml) (**B,D,F**) or LPS (50 ng/ml) (**C,E,G**) treatment for 2,4 and 6 hours. Phagocytic index expressed as ratio of relative fluorescence units (FITC/DAPI) expressed as % from vehicle control. Bars illustrate mean of three independent experiments ± SE (student’s t-test ***p < 0.001, **p < 0.01, *p < 0.05-significance to respective vehicle control).

Our initial findings using fluorometric analysis indicates that chronic morphine treatment inhibits phagocytosis of opsonized heat killed *E. coli, S. aureus* as well as opsonized dextran bead (Fig. 1A). Interestingly, morphine’s attenuation of phagocytosis was more significant following 30 minute incubation of Gram-positive heat killed opsonized (HKO) *S.aureus,* and 60 min incubation of opsonized dextran beads (OPDex beads) (40% inhibition) than of Gram-negative HKO *E. coli* (20% inhibition) (Fig. 1A). In order to examine differential effects of gram positive and gram negative bacterial products on morphine induced inhibition of phagocytosis of Gram-positive and Gram-negative bacteria we examined the effects of lipoteichoic acid (LTA) (component of Gram-positive bacteria) a TLR2 ligand, and a lipopolysaccharide (LPS) (component of Gram-negative cell wall) a TLR4 ligand on phagocytosis.

TLR2 receptors are activated by series of ligands from Gram-positive pathogens. Lipoteichoic acid (LTA) is a major constituent of the cell wall of Gram-positive bacteria and it binds to TLR2/TLR6 heterodimer. Activation of TLR2 via LTA (10 ng/μl) lead to sustained inhibition of phagocytosis of HKO *E. coli* (Fig. 1B) particles, OPDex bead (Fig. 1D), HKO *S. aureus* particle (Fig. 1F), in vehicle treated but not in morphine treated cells. The inhibitory effects of morphine and LTA are neither additive nor synergistic. Similar observations were noted in macrophages phagocytizing OPDex bead following chronic morphine treatment and TLR2 activation with Peptidoglycan from *S. aureus* (PGN- 80 μg/ml) (Supplemental Fig. 1A).

After undergoing chronic morphine treatment (1 μM overnight), J774 cells were treated with LPS (50 ng/ml) for 2, 4 and 6 hours prior to bacterial incubation. As expected, morphine alone inhibited, while LPS alone increased phagocytic index in a time dependent manner. Surprisingly J774 cells treated with morphine and LPS (morphine+LPS) potentiated the increase in phagocytosis compared to LPS alone or vehicle control (Fig. 1C,E,G) contrasting the findings observed in cells undergoing morphine treatment alone. This effect was observed in cells exposed to HKO *E.coli* (Fig. 1C), HKO *S. aureus* for 30 minutes (Fig. 1G), as well as following 60 minutes of exposure to opsonized dextran bead (OPDex bead) (Fig. 1E). This indicates that activation of TLR4 receptor in cells pretreated with morphine leads to increase in phagocytosis.

**Figure 2:**
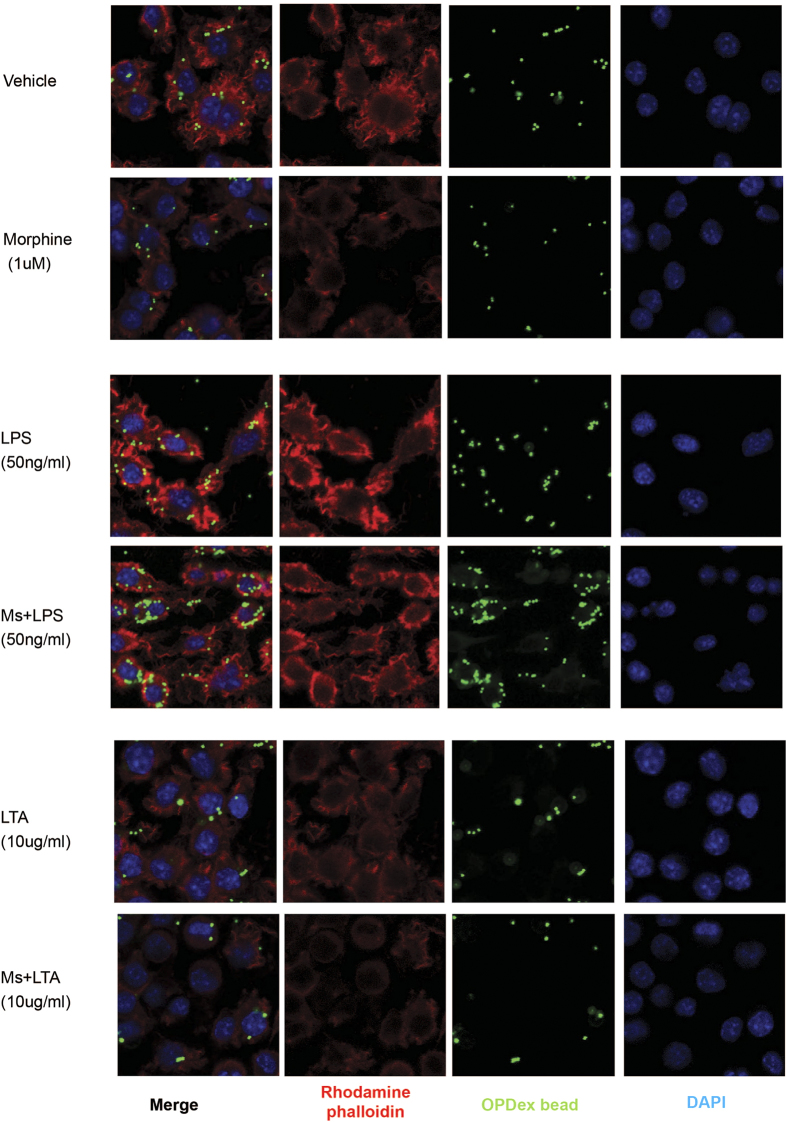
Microscopic analysis of morphine modulation of phagocytosis following TLR4 and TLR2 activation. Cells undergoing chronic morphine treatment (1 μM overnight) were exposed to (**A**) vehicle, (**B**) LTA (10 μg/ml) or (**C**) LPS (50 ng/ml) 4 hours prior to phagocytosis. Following internalization of FITC conjugated OPDex bead (1:20, cell:bead ratio) for 60 min, cells were washed with trypan and PBS, fixed with paraformaldehyde, permeabilized with acetone and stained for f-actin using rhodamine phalloidin (red), and DAPI (blue). Images illustrate confocal microscopic analysis at 60× with additional digital magnification.

Confocal microscopic analysis of J774 cells undergoing morphine treatment in presence of TLR ligands (Fig. 2) further confirmed the previous findings indicating LTA treatment leads to inhibition of phagocytosis in vehicle and morphine treated cells. As expected, morphine treatment alone led to inhibition of phagocytosis, while increased actin polymerization and potentiation of OPDex bead phagocytosis was seen in LPS treatment alone. These effects were further potentiated in the presence of morphine.

**Figure 3:**
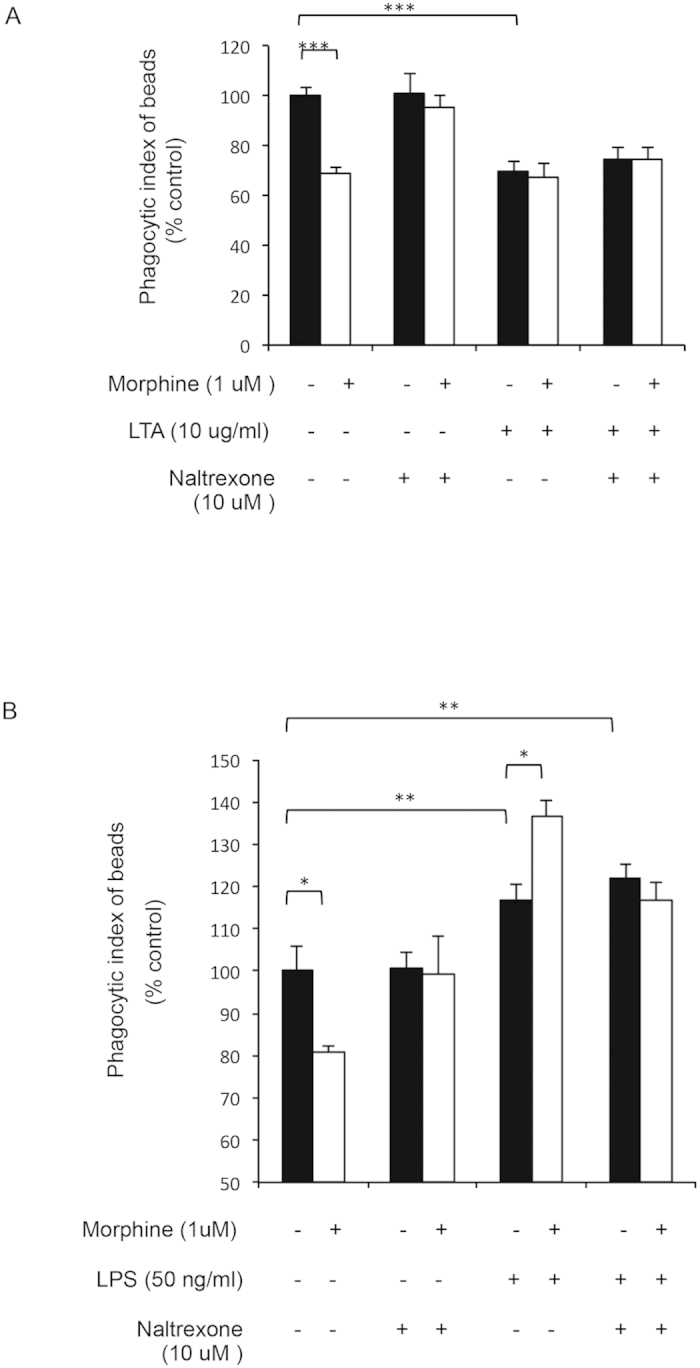
Modulation of phagocytosis following activation of TLR 4 and TLR 2 in morphine treated primary macrophages is naltrexone reversible. Fluorometric quantification of phagocytosis by J774 macrophages. Naltrexone (10 μM) was added 2 hr prior to overnight morphine (1 μM) treatment of primary macrophages from WT mice. (**A**) LTA (10 μg/ml) and (**B**) LPS (50 ng/ml) were added 4hr before addition of OPDex beads. Phagocytosis was allowed for 60 min and cells were analyzed via fluorometric analysis and expressed as % control (Phagocytic index = FITC (RFU)/DAPI(RFU)). Data quantified as % from vehicle control, mean ± standard error. Significance was determined using student’s t-test (***p < 0.0001, **p< 0.001, *p < 0.05).

**Figure 4:**
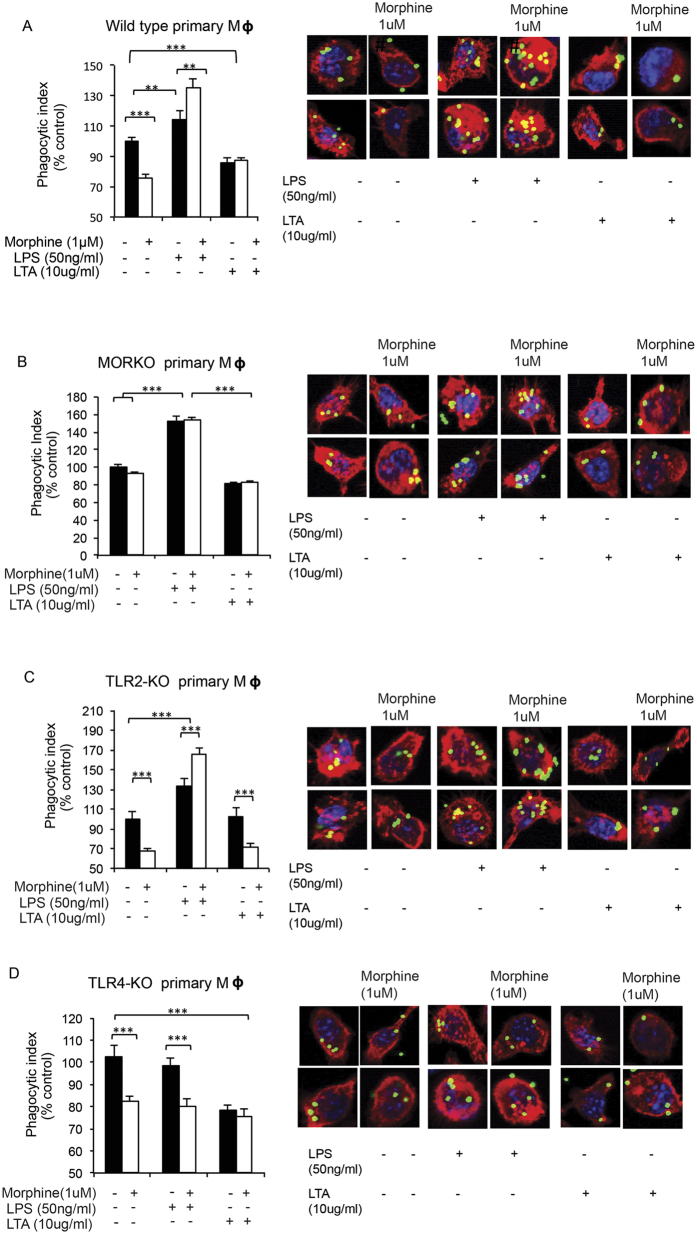
Effects of chronic morphine and TLR ligands in knock-out mice. Macrophages extracted from (**A**) wild-type, (**B**) MORKO, (**C**) TLR2-KO and (**D**) TLR4-KO were treated *ex vivo* with morphine, LPS and LTA as previously described for 4 hours, and analyzed using fluorometric assay and confocal microscopy (single cells) following 60 min of phagocytosis with OPDex bead (***p < 0.0001).

To further establish the role of opioid receptors in this process we utilized naltrexone, a known opioid receptor antagonist. Morphine-induced inhibition of phagocytosis was abolished in the presence of naltrexone (Fig. 3). LTA treated cells maintained their inhibition in the presence of naltrexone and morphine (Fig. 3A), suggesting that inhibitory effects of LTA and morphine on phagocytosis are independent and are neither additive nor synergistic. Morphine induced increase in phagocytosis following LPS treatment was abolished in cells pretreated with naltrexone (Fig. 3B), indicating that morphine exerts its effect on FcγR mediated phagocytosis in J774 macrophages through the classical opioid receptor. Since morphine has a high affinity for μ opioid receptor (MOR), we wanted to examine the role of MOR in these processes using primary macrophages from wild-type (WT) and μ-opioid receptor knock out (MORKO) mice. Similar to the observations made with J774 macrophages, in primary peritoneal macrophages from WT mice treated *ex vivo* with, morphine alone and morphine+LTA, decreased, while morphine+LPS, increased, phagocytosis (Fig. 4A). Similar to our naltrexone studies, effects of morphine on differential modulation of phagocytosis were abolished in primary macrophages from MORKO mice treated *ex vivo* with morphine, LPS and LTA (Fig. 4B).

Taken together these results indicate that morphine treatment alone inhibits phagocytosis, with inhibition being greater for gram positive bacteria compared to gram negative bacteria. Gram negative (LPS) and gram positive (LTA) bacterial products have differential effects on morphine induced inhibition of phagocytosis. LPS potentiated phagocytosis in chronic morphine treated macrophages more than LPS alone, through a MOR dependent mechanism whereas LTA inhibited phagocytosis. The inhibitory effects of LTA on macrophage phagocytosis are independent of morphine and are neither additive nor synergistic.

### TLR2 activation by LTA priming decreases phagocytosis in primary macrophages

To examine the role of TLR2 in LTA mediated inhibition of phagocytosis we utilized primary peritoneal macrophages from TLR2KO mice. Murine peritoneal macrophages were extracted from TLR2KO mice and treated with morphine, LPS and LTA *ex vivo* as previously described.

**Figure 5:**
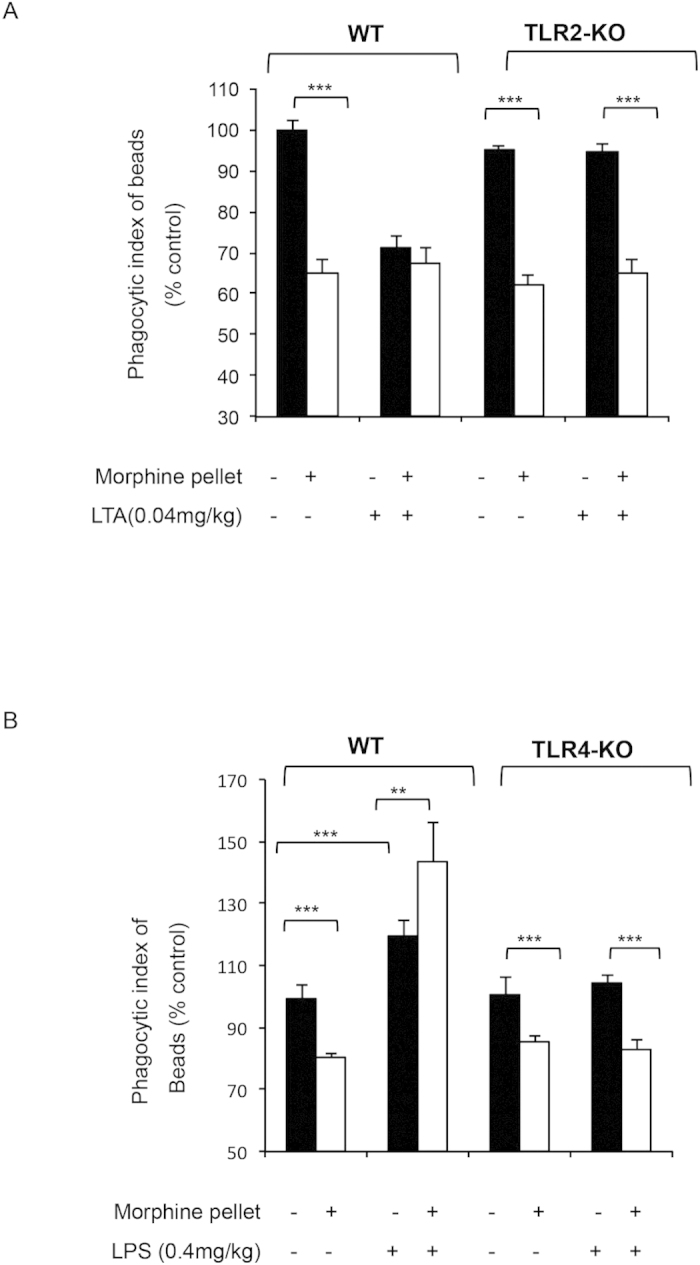
Effects of chronic morphine and TLR ligands (LPS and LTA) in knock-out mice. Fluorometric analysis of *ex vivo* phagocytosis by primary macrophages from WT, TLR2-KO or TLR4-KO mice treated *in vivo* with morphine (75 mg slow releasing pellet for 3 days), and injected IP with LTA (**A**) or LPS (**B**) for 4 hours prior to sacrifice and cell harvesting. Phagocytosis was assessed *ex vivo* following 60 min of OPDex incubation, using fluorometric analysis and expressed as % control (Phagocytic index = FITC (RFU)/DAPI(RFU). Data quantified as % from vehicle control, mean ± standard error. Significance was determined using student’s t-test (**p < 0.001, ***p < 0.0001).

Similar to *in vitro* studies LTA-mediated TLR2 activation, as well as morphine treatment led to inhibition of phagocytosis in primary macrophages from WT mice (Fig. 4A). In macrophages from TLR2 KO mice (Fig. 4C), morphine mediated inhibition of phagocytosis was sustained while LTA effect was abolished indicating that TLR2 was essential in this process and that LTA effects are independent of morphine. Similar effects were observed in macrophages extracted from WT and TLR2KO mice undergoing morphine and LTA treatment *in vivo* (Fig. 5A). LPS treatment was unaffected in the absence of TLR2 receptor confirming that LPS acts solely through the TLR4.

Taken together these data indicate that morphine through activation of classical opioid receptors (namely MOR) inhibits phagocytosis. Similarly, activation of TLR2 by LTA also inhibits phagocytosis. Although both LTA and morphine lead to inhibition of internalization, their effects are independent and are neither synergistic nor additive. The possible explanation could be that individual treatment might be reaching maximal inhibition and causing a ceiling effect.

### Morphine increases phagocytosis following TLR4 activation in primary macrophages

To further elucidate the effects of TLR on phagocytosis following chronic morphine treatment we utilized TLR2 and TLR4 knockout (KO) mice. In primary macrophages from TLR4 KO mice same level of inhibition was observed in cells treated with morphine alone and morphine+LPS. Therefore, absence of TLR4 led to loss of LPS mediated amplification of phagocytosis, causing morphine+LPS to display only morphine mediated inhibition and not potentiation as seen in the primary macrophages from WT mice (Fig. 4D). As expected, LTA and morphine maintained same levels of inhibition of phagocytosis in TLR4 KO mice (Fig. 4D) as seen in macrophages from WT mice undergoing the same treatment (Fig. 4A), since LTA and morphine do not require TLR4 for signaling.

These data were also confirmed using *in vivo* studies, following implantations with slow release morphine or placebo pellets (72 hours), and LPS injections (0.4 mg/kg) 4 hours prior to sacrifice. Peritoneal macrophages were collected via peritoneal lavage and were treated with OPDex bead for 60 minutes. Similar to previous experiments, *in vivo* morphine and LPS treatment result in increased phagocytosis in WT mice while in TLR4 KO mice, LPS effect was abolished while morphine-mediated inhibition was maintained (Fig. 5B).

Taken together these data indicate that TLR4 activation by morphine is essential for mediating increase in phagocytosis induced by LPS priming in chronic morphine treated macrophages.

### Morphine modulates TLR expression

Transcriptional responses to TLR activation modulate phagocytosis^18^,^19^. Activation of TLR4 by LPS has been implicated in enhancement of phagocytosis by upregulation of scavenger receptor A and activating Fc gamma receptors^25^,^26^. Short term LPS exposure (4hrs) used in this model was not able to induce expression changes in FcγR1 (Supplemental Fig. 1B). Furthermore, short-term morphine exposure (1 μM for 4hr) in the presence of LPS (50 ng/ml for 4 hours) was unable to induce morphine mediated increase in phagocytosis (Supplemental Fig. 1C). Although, as expected LPS increased phagocytosis of both morphine and vehicle treated cells, morphine maintained its inhibition of internalization.

**Figure 6:**
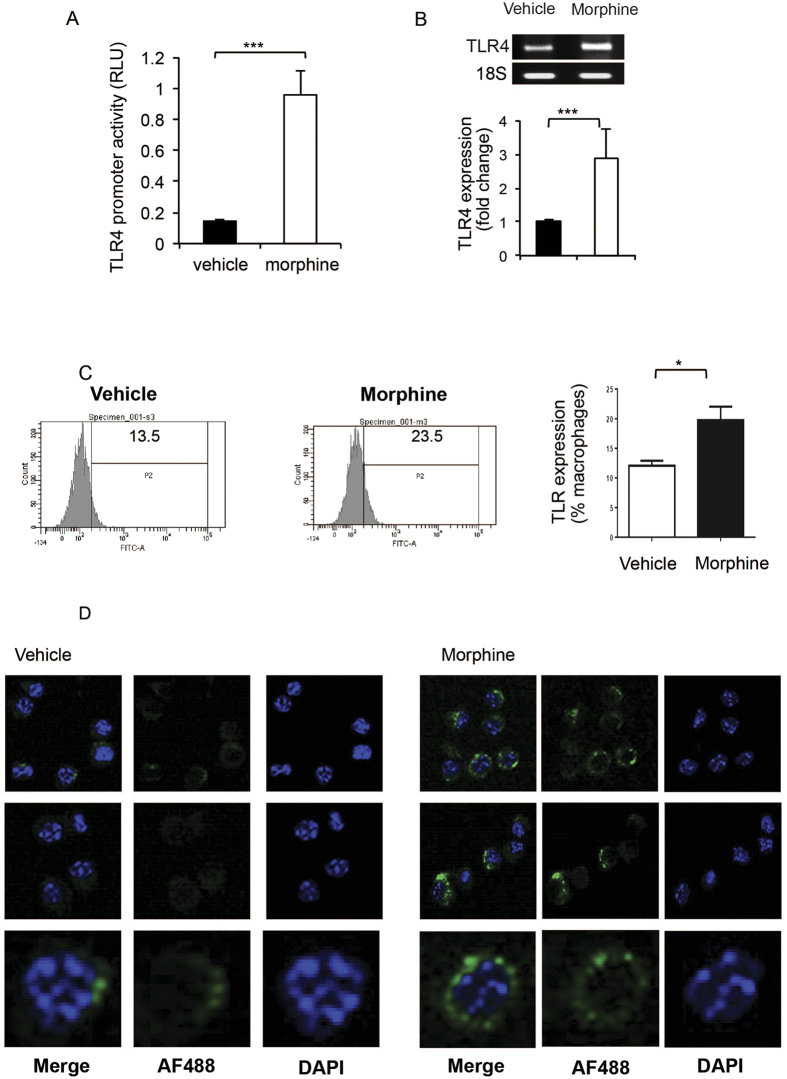
Chronic morphine treatment increases TLR-4 expression. (**A**) TLR4 promoter activity as measured by luciferase. J774 cells were transfected using Fugene HD transfection reagent (roche) with a plasmid containing firefly luciferase, driven by the TLR4 full-length (~2.2 kb) promoter. Chronic morphine treatment was started 24 hours post-transfection. Promoter activity was measured by luciferase activity normalized to renilla luciferase values and represented as Relative Luciferase Units (RLU). (**B**) QT-PCR analysis of TLR4 expression following overnight 1 uM morphine treatment. (**C**) After undergoing chronic morphine (1 μM) treatment overnight J774 cells were collected, fixed and stained with Anti-Mouse CD284 (TLR4) Alexa Flour 488 antibody (eBioscience; cat# 53-9041-80) for 30 min at 4 °C and analyzed using Diva software (BD Bioscience). Data quantified as % positive cells, mean ± standard error. Significance was determined using student’s t-test (*p < 0.05, **p < 0.001, ***p < 0.0001). (**D**) Confocal microscopy analysis of the cytospin from the cells collected as in (**C**). Images are 4.2 times zoomed after collecting at 40×.

Since only prolonged, chronic morphine treatment augments LPS induced increase in phagocytosis we set out to examine if chronic morphine treatment modulates expression of TLR4. Initially, TLR4 promoter driving a luciferase expression reporter construct was utilized to examine TLR4 promoter activity. Following transfection with the reporter plasmid, J774 cells were treated overnight with 1 μM morphine. Next day cells were quantified for luciferase expression. Morphine treatment significantly increased TLR4 luciferase-reporter activity compared to the vehicle control (Fig. 6A). Next, we examined mRNA levels for TLR4 using QT-PCR. Our results show that chronic morphine treatment lead to significant increase in TLR4 message levels (Fig. 6B). Similar findings were observed with surface expression of TLR4 quantified via FACS analysis(Fig. 6C) and confocal microscopy (Fig. 6D), indicating that morphine treatment alone increased cell surface expression of TLR4.

Together these data establishes that morphine increases TLR4 message and protein expression levels on J774 macrophages and is responsible for the potentiated effects of LPS treatment on phagocytosis in chronic morphine treated macrophages.

### Modulation of phagocytosis of live bacteria by chronic morphine treatment following LPS and LTA priming

**Figure 7:**
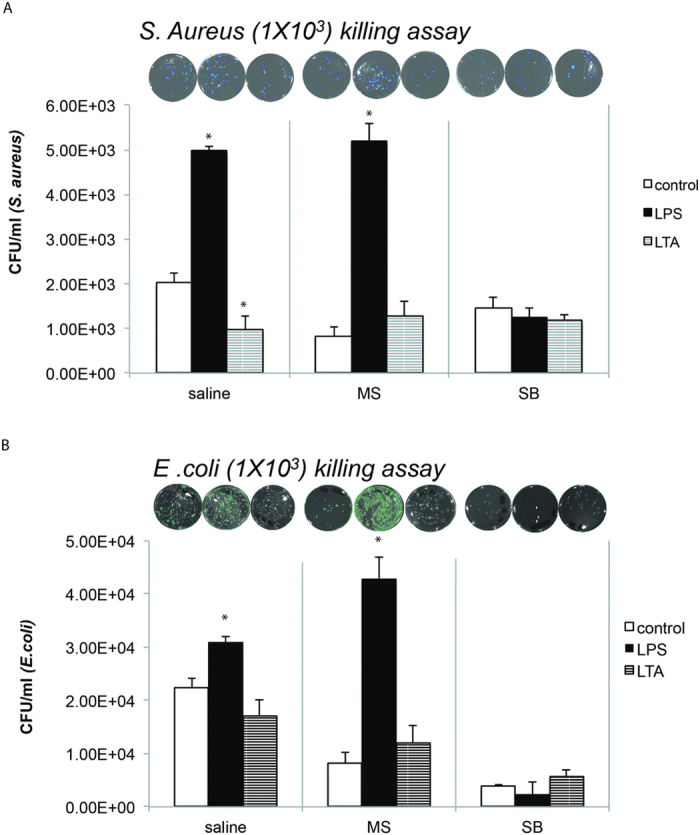
Modulation of phagocytosis of live bacteria by chronic morphine treatment following LPS and LTA priming: J774 macrophage cells were pretreated with morphine (1 um) overnight and than exposed to either vehicle, LPS (50 ng/ml) or LTA (10 ug/ml) for 4 hours and then incubated with either live GFP-tagged E. coli (1 × 10^3^) (**A**) or live luciferase tagged S. aureus (1 × 10^3^) , bioluminescent strain 100) (**B**). Cell to bacteria ratio was maintained at 1:20. To determine the mechanism underlying increase in phagocytosis by morphine treated LPS, J774 macrophages were treated with p38 MAP kinase inhibitor (SB 202190-10 uM) for 4 hours and then treated with morphine overnight and then incubated with live bacteria as described above. Representative bioluminescence photographs (upper insert) of bacterial culture plates after overnight culture and histograms represents mean±SE of colony-forming units (CFUs) of S. aureus/E. coli (lower panel) recovered after lysing of J774 macrophage cell line (0.2 × 106/200 μl). *p< 0.05 (student T test).

To investigate if morphine treatment inhibits phagocytosis with live gram (+) and gram (−) bacteria, J774 macrophage cells were pretreated with morphine (1 um) overnight as described in studies carried out in Fig. 1. Cells were than incubated with either Vehicle, LPS or LTA for 4 hours and then incubated with either live GFP-tagged E. coli (1:20) (Fig. 7A) or live luciferase tagged S. aureus (bioluminescent strain 100) (Fig. 7B). Morphine treatment alone inhibited phagocytosis of both S. aureus (Fig. 7A) and E. coli (Fig. 7B). Treatment with LPS potentiated phagocytosis of both gram (+) and gram (−) live bacteria, similar to what was observed with opsonized heat killed bacteria in Fig. 1. Similarly, treatment with LTA inhibited phagocytosis of both gram (+) and gram (−) bacteria. To determine the mechanism underlying increase in phagocytosis by morphine treated LPS primed macrophages, we pretreated J774 macrophages with p38 MAP kinase inhibitor (SB 202190) as described in the method section. Treatment of macrophages with p-38 MAP kinase inhibitor, inhibited both LPS and morphine induced increase in phagocytosis of both gram (+) and gram (−) bacteria (Fig. 7A,B) indicating that both TLR4 and mu-opioid receptor induced increase in phagocytosis is mediated by activation of the p38 MAP kinase pathway. To determine if incubation with the respective bacteria resulted in compromised cell viability, cell counts were evaluated using trypan blue exclusion method in the absence and presence of bacteria. Our results show that bacterial incubation did not significantly alter cell viability (supplementary Fig. 2).

### Differential modulation of bacterial killing

Effective bacterial clearance consists of two steps: internalization and bacterial killing. TLR signaling can modulate ROS production, a key step in bacterial killing^27^. Since, we noted differential rates of internalization in the presence of TLR ligands and chronic morphine treatment, experiments were designed to examine the effects of morphine in the context of LPS and LTA on bacterial killing following phagocytosis.

**Figure 8:**
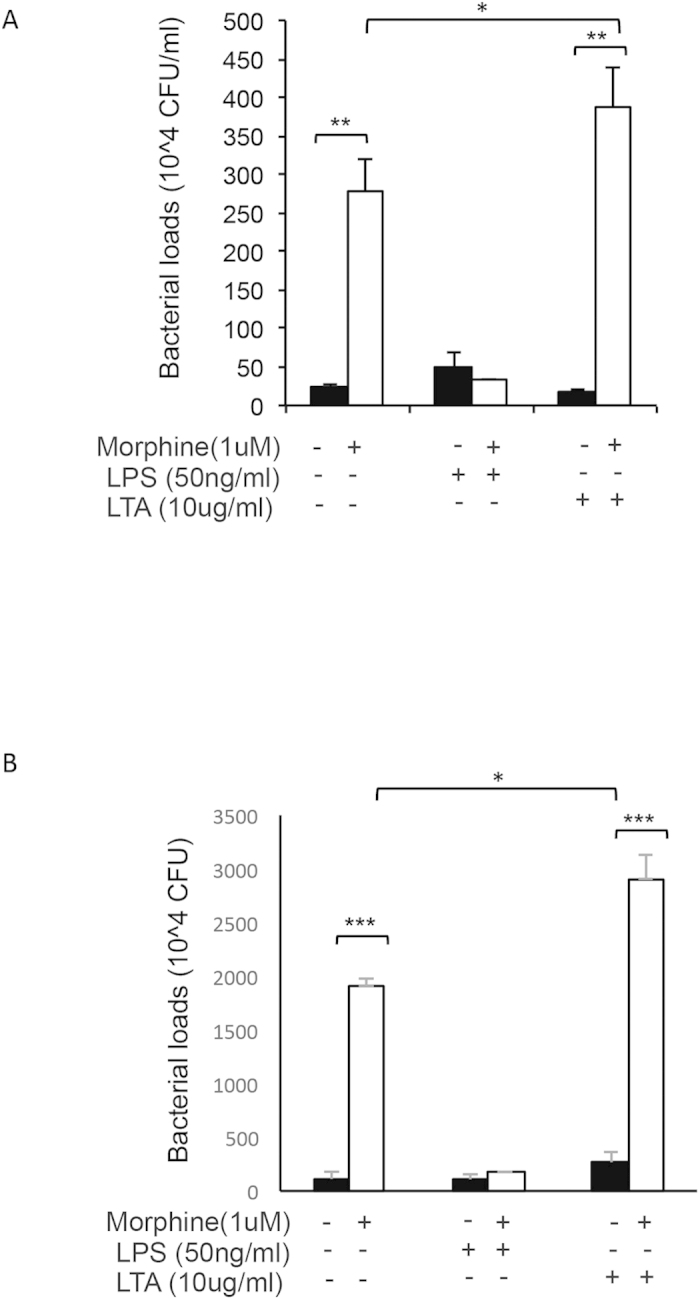
LPS and LTA in presence of morphine differentially modulates bacterial killing. J774 cells undergoing morphine treatment (1 μM overnight), were treated with LPS (50 ng/ml) or LTA (10 μg/ml) for 4 hours (in antibiotic free media) prior to addition of GFP *E. coli* (**A**) and *S. pneumoniae* (**B**). Following 30 min of phagocytosis cells were washed four times with PBS containing 1% penicillin/streptomycin and then washed with antibiotic free medium and incubated overnight in antibiotic free media containing morphine, LPS or LTA (where appropriate). Next day cells were lysed and plated on LB plates (Ampicillin 100 μg/ml) for GFP *E. coli* and Kanamycin (50 ug/ml) blood agar plates for *S. pneumoniae,* and evaluated for bacterial colony formation (CFU) the next day. Significance was determined using student’s t-test (*p < 0.05, **p < 0.001,***p < 0.0001).

In these studies, we examined macrophage bactericidal capabilities following morphine, LPS, LTA treatment (as previously described). Following 30 minutes of phagocytosis of live *E. coli* (wild type) and *S. pneumoniae,* J774 cells were washed four times in antibiotic containing PBS to eliminate proliferation and growth of non internalized bacteria. Cells were then incubated in antibiotic-free media for 24 hours to allow killing of internalized bacteria. The next day cells were homogenized and plated on *with their respective resistant antibiotic,* LB Ampicillin growth plates for GFP *E.coli* and *Kanamycin* blood agar plates for *S. pneumonia*. Intracellular bacterial growth was quantified by the colony forming units (CFU) counted the next day and data was reported in Fig. 8. Although morphine treatment alone inhibited phagocytosis of bacteria, the number of viable bacteria in cells treated with morphine was significantly greater suggesting morphine treatment led to greater intracellular bacterial growth thus indicating a significant inhibition of bacterial killing. LPS treatment led to increased internalization in morphine and vehicle treated cells, however subsequent bacterial killing was greatly potentiated as evident by the comparable bacterial loads between the vehicle and LPS treatment. Interestingly LTA treatment led to inhibition of internalization and had no effect on bactericidal ability by itself. However, following morphine+LTA treatment, as was observed previously, internalization was significantly lower, however bacterial killing was compromised with significantly greater bacterial viability compared to all other treatment groups.

**Figure 9:**
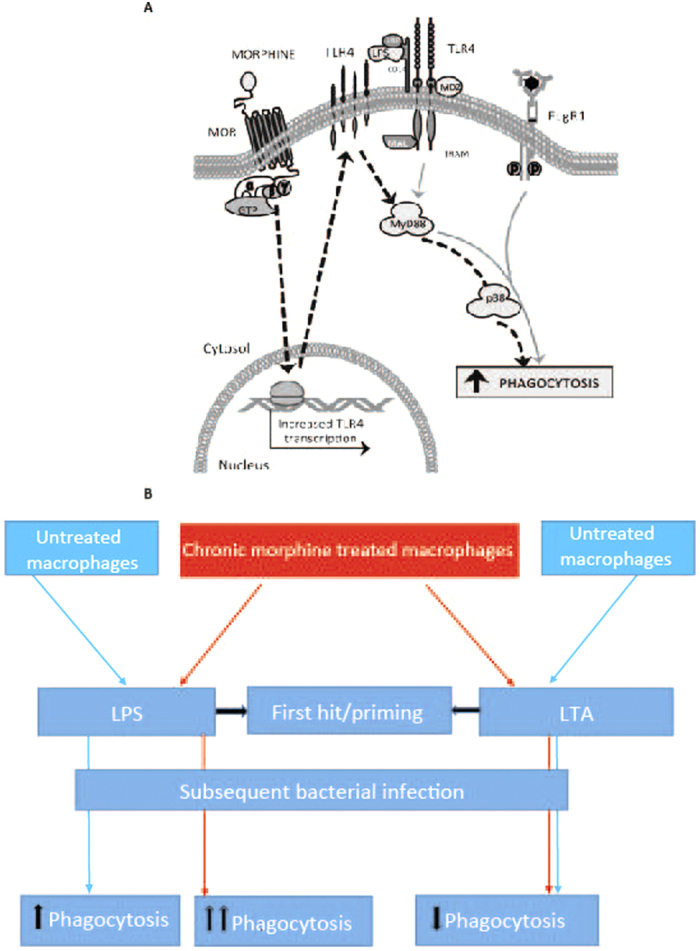
(**A**) Role of MOR and TLR in modulation of FcγR mediated phagocytosis. Morphine via activation of MOR increases TLR4 expression. In presence of LPS, TLR4 is activated to turn on MyD88 and p38 MAPK. By p38 MAPK phosphorylation, actin polymerization in increased leading to increased phagocytosis. (**B**) Schematic showing differential effects of LPS and LTA priming on morphine induced inhibition of phagocytosis. Chronic morphine treatment decreases phagocytosis in macrophages. Priming with LPS, increases phagocytosis in morphine treated macrophages which is greater than the effects of LPS alone. This potentiation is mediated by increase in TLR4 message and surface expression by morphine. LTA priming is associated with inhibition of phagocytosis which is independent to morphine’s effects and is neither additive nor synergistic.

These results indicate that bacterial killing is potentiated with LPS-mediated TLR4 activation, and inhibited in LTA-mediated TLR2 activation in cells undergoing chronic morphine treatment (Fig. 9).

## Discussion

This study is the first to demonstrate the differential effects of LPS and LTA on morphine induced inhibition of macrophage phagocytosis and bacterial clearance. LPS treatment leads to increased phagocytosis in morphine treated macrophages that is higher than effects of LPS alone. Morphine treatment increase TLR4 message and protein levels thereby potentiating LPS induced phagocytosis. Several lines of evidence support these findings. First, that chronic morphine treatment alone inhibits, while in the presence of LPS, morphine potentiates FcγR mediated phagocytosis. Second, this effect is abolished in TLR4 KO but not in TLR2 KO mice, indicating that LPS is acting solely through the TLR4 receptor. We further demonstrate that chronic morphine treatment upregulates TLR4 expression. LTA, however inhibits phagocytosis with its inhibitory effects being independent of morphine induced inhibition and are neither additive nor synergistic.

Morphine-mediated suppression of innate and adaptive immunity is manifested through high frequency of bacterial infections in opioid users and abusers. Macrophages play a key role in morphine mediated modulation of innate and adaptive immunity, and are essential in elimination of bacterial infections. Although many groups have reported morphine modulation of macrophage functions such as phagocytosis and inflammation, to our knowledge there have been no studies examining morphine’s effect on the crosstalk between the inflammatory TLR, phagocytic FcγR and opioid receptors. Due to the significant role that macrophages play in pathogen clearance as well as deleterious effects that occur as a result of disruption of macrophage function, we set out to explore this gap in knowledge and address mechanisms by which morphine modulates TLR signaling and FcγR mediated phagocytosis.

Our previous work and present data shows that chronic morphine treatment inhibits FcγR mediated phagocytosis^28^. Studies have shown the effects of LPS on phagocytosis and found an increase in phagocytosis of sheep RBCs by bone marrow-derived mouse macrophages^21^ and increase in phagocytosis of peritoneal macrophages by TLR4 activation in peritoneal sepsis model^22^. This observation is further validated in studies that show LTA inhibits *S. aureus* phagocytosis and killing by human polymorphonuclear leucocytes in concentration dependent manner^24^. We found that following short-term LPS activation of TLR4 in the context of opioid treatment lead to increased phagocytosis by J774 macrophages as well as by primary macrophages treated *in vitro* and *in vivo*. Morphine+LPS enhancement of phagocytosis is abolished in macrophages pretreated with naltrexone, as well as primary macrophages from TLR4 KO mice and MORKO mice. Data indicates that morphine+LPS increase in internalization is mediated by classical opioid receptors such as MOR as well as by the TLR4, and that expression and activation of both receptors is required for the amplification of phagocytosis. On the other hand, activation of TLR2 receptor by LTA lead to inhibition of phagocytosis bringing down the phagocytic index to the same values as cells undergoing chronic morphine treatment. However, this inhibition was independent of morphine induced inhibition and is neither additive nor synergistic, which is probably because of maximal inhibition by morphine causing the ceiling effect. This was true for macrophage cell line J774 as well as *in vitro* treated macrophages from WT mice, while in primary macrophages from TLR2 KO mice as expected, this effect of LTA was abolished.

TLRs activate transcription of a large number of genes whose gene products are known to participate at all stages during phagocytosis, ranging from microbial recognition, actin cytoskeletal dynamics, membrane trafficking, ion transport, proteolysis, and antigen presentation^29^,^30^. Our data shows that chronic morphine treatment by upregulating TLR4 message and surface expression, amplifies LPS mediated TLR4 activation leading to increased phagocytosis and bacterial killing. Our group and others have explored LPS-induced inflammation and accelerated progression to septic shock seen with chronic morphine exposure. Morphine has been known to enhance LPS-induced leukocyte-endothelial adhesion, elevated IL-1β, TNF-α, and IL-6 serum levels^31^. Therefore it is not surprising that morphine amplifies TLR4 activation and through that mechanism enhances macrophage phagocytosis.

There has been some controversy in the literature as to LPS ability to activate both TLR2 and TLR4^32^. Several groups report that LPS can activate TLR2, while others claim that LPS is a standard TLR4 ligand^33^. Although, this controversy has not yet been fully resolved, our model confirms that LPS used in this study only activates TLR4, since in our studies utilizing primary macrophages from TLR4-KO mice we note that in absence of TLR4, LPS and morphine+LPS amplification of phagocytic index is abolished while LTA and morphine maintained their inhibitory effect. In macrophages from TLR2-KO mice, morphine+LPS increase in phagocytosis was as robust as in WT, indicating that morphine+LPS had no effect on TLR2 activation.

Although chronic morphine treatment inhibits phagocytosis of Gram-positive and Gram-negative bacteria, morphine treatment leads to a greater inhibition of phagocytosis of Gram-positive bacteria. This is in part due to the fact that LPS, a component of Gram negative bacterial cell wall and a TLR4 ligand leads to increased phagocytosis in presence of morphine by increasing the internalization of the pathogen by activation of TLR4 which has been upregulated by morphine. On the other hand LTA, a component of Gram-positive bacteria, activates TLR2 leading to inhibition of phagocytosis while maintaining morphine’s inhibitory effect, which is independent. LTA induced inhibition is neither additive nor synergistic with morphine. Since we claim that TLR4 activation in presence of morphine has a dramatic increase in phagocytosis, one would expect to see an increase of phagocytosis of Gram-negative bacteria and their high LPS content. However, in presence of morphine we see an inhibition of phagocytosis of both Grams–positive and Gram-negative heat-killed opsonized bacteria. This finding can be explained by the brief incubation time of cell and bacteria in this model. Macrophages are exposed to HKO *E. coli* for maximum of 30 minutes which is insufficient amount of time to induce the same effect as the 4 hour pretreatment with LPS. On a systemic level, one would expect to see an increase in phagocytosis since during an infection bacterial growth and macrophage phagocytosis are long-term processes which would resemble the effects we see in 4 hour pre incubation with LPS.

Our study testifies to immune activation in presence of morphine and LPS which translates clinically to a gram negative infection in a chronic opioid abuser/user. An opioid addict or chronic opioid user who gets gram negative infection has chances of potentiated phagocytosis on subsequent infection. Thus, it raises a very important clinical question of using low dose LPS priming in chronic opioid users and abusers as a prophylactic measure to prevent serious infections. There is a need of well conducted clinical studies to answer this question. Also, it is important to note that infection with gram positive bacteria in opioid abuser would call for aggressive management in terms of providing antibiotic coverage since such patients could be prone to a worse clinical outcome with a second infection, because of impaired phagocytosis.

Morphine mediated immunosuppression is a complex and important process. As opioid prescription pain relievers continue to be prescribed as the standard therapies for pain management, and as the opioid abuse continues to rise, it is important to further understand these mechanisms in order to identify targets necessary for development of better, less immunosuppressive pain management strategies.

## Methods

### Reagents

Heat killed *E. coli* (E2861*- Escherichia coli* (K-12 strain) BioParticles, fluorescin conjugate (ex 494/em518), heat killed *S. aureus* (S-2851*-Staphylococcus aureus* (Wood strain without protein A) BioParticles^®^, fluorescein conjugate (ex 494/em518), respective opsonizing reagents (E2870 - *E. coli*, S-2860 - *S. aureus*), dextran beads (yellow-green fluorescent Fluor-Spheres (F8852 Invitrogen; ex488nm/518 nm) and DAPI were obtained from Invitrogen. Morphine-HCl powder as well as 75 mg slow release pellets were a generous gift from NIDA (National Institute of Drug Abuse). Lipopolysaccharides from *Escherichia coli* 055:B5 (cat# L2880) and Lipoteichoic acid from Staphylococcus aureus (cat# L2515) were obtained from Sigma Aldrich. GFP E.coli (gift from Dr. Sundaram Ramakrishnan), *Streptococcus pneumoniae* (*S. pneumoniae*) serotype 3 was obtained from ATCC (cat# 6303; Rockville, MD). *S. pneumoniae* was grown overnight at 37 °C by streaking onto a blood agar plate (Becton, Dickinson and Co.). The colonies were picked, inoculated into brain heart infusion (BHI) broth and incubated for 6 h at 37 °C to produce log-phase organisms. The concentration of bacteria was determined spectrophotometrically (OD590) and confirmed by plating onto blood agar plates.

### Cells

Murine macrophage cell line J774.1 was obtained from ATCC, and cultured in DMEM media (GIBCO) supplemented with 10% heat-inactivated fetal bovine serum (HyClone), and 1% Penicillin/Streptomycin (GIBCO). Cells were grown at standard growth conditions.

### Animals

All animals were housed in pathogen-free facilities and all procedures were approved by the University of Minnesota Institutional Animal Care and Use Committee (IACUC) and were performed in accordance with applicable IACUC guidelines. MORKO mice (C57BL/6 × 129/Ola genetic background) were produced as described previously by Loh and colleagues^34^. Briefly, a *Xho*I/*Xba*I fragment, which spans the entire exons 2 and 3, was replaced with a Neo^r^ cassette followed by the ligation of a thymidine kinase expression cassette to the 3′ end of this segment. WT mice (B6129PF1/J) and C57BL/6, 8 weeks of age, were obtained from The Jackson Laboratory (Bar Harbor, ME). TLR2 KO and TLR4 KO C57BL/6 background mice were a generous gift from Dr. J. Ohlfest, and are commercially available through Jackson laboratories (strain # C.C3-*Tlr4*^*Lps-d*^/J stock number 002930, and *Tlr2* - B6.129**-***Tlr2*^*tm1Kir*^/J stock number 004650). Animal studies have been reviewed and approved by University of Minnesota Institutional Animal Care and Use Committee.

### Chronic morphine administration

For all *in vitro* experiments 1 μM morphine HCl was added overnight (18 hours). For studies involving morphine treatment *in vivo,* mice were implanted with 75 mg slow release morphine/placebo pellets (NIDA) for 72 hours. During the extraction of peritoneal cells from morphine treated WT or MORKO mice, 1 μM morphine was maintained in all PBS and media used in the experiment in order to prevent withdrawal. Concentrations used in the *in vitro* paradigm were chosen to closely replicate morphine plasma levels (11 ng/ml-1440 ng/ml) which are present in patients receiving morphine for moderate to chronic pain (2.5 mg–90 mg every 4 hours), similar to what is observed in mice following 72 hours implantation with 75 mg morphine pellets^35^.

### Fluorometric assay

Cells were plated at 10,000/100ul of media per well of a 96-well plate, treated with morphine and cultured overnight in standard conditions (37 °C, 5% CO_2_, 60% rh). The following day, opsonized dextran beads, opsonized heat killed *E. coli* and opsonized heat killed *S. aureus* were added (in 1:20, cell:bacteria/bead ratio). Opsonization was done with IgG opsonizing reagent (Invitrogen) for 1 hour in 37 °C, according to the manufacturer’s instructions. The reaction was stopped at different time points by addition of trypan blue, which quenches fluorescence of non-internalized particles. Cells were washed two times and stained with DAPI. Fluorescence was recorded using a fluorescence plate reader (BMG; FLUOstar Omega) at ex485/em520 (FITC), and ex355/em460 (DAPI). Data was quantified as Phagocytic Index = FITC (Relative Fluorescence Units (RFU))/DAPI(RFU), indicative of particle fluorescence/cell. The y-axis was normalized to fluorescence of the vehicle treatment with the vehicle control and data was expressed as percentage of vehicle control (% control).

### Confocal microscopy

Following the morphine, LPS and LTA treatments as described above, cells were allowed to phagocytose opsonized dextran beads and stained with rhodamine phalloidin according to manufacturer’s instructions. Images were taken using Nikon inverted confocal microscope (model: Ti-E eclipse 100) and Roper camera (model: Cool-snap HQQ) at 60× with additional digital magnification. Images shown are the flattened sum of 15 cross sections.

### TLR4 reporter assay

J774 cells were transfected with a plasmid (pGL3 backbone; kindly provided by Dr Thierry Roger), where firefly luciferase was driven by the TLR4 full-length (~2.2 kb) promoter, using Fugene HD transfection reagent (roche). In all cases, constitutively expressing renilla luciferase was co-transfected as transfection control. 1 μM Morphine treatment was started 24 hours post-transfection to establish the chronic paradigm. On completion of the treatment, cells were washed with PBS and luciferase activity was measured using the Dual-Luciferase assay kit (Promega) as per the manufacturer’s protocol. The luciferase activity was normalized to renilla luciferase values and represented as Relative Luciferase Units (RLU).

### PCR

Following overnight morphine treatment cells were washed and collected in TRIzol reagent. RNA was isolated using TRIzol reagent according to the manufacturer’s protocol (Invitrogen). cDNA was synthesized using 2 ug of total RNA, 10X Random primers and RNase MMLV reverse transcriptase according to manufacturer’s specifications (Promega). All samples were run in triplicates and normalized to 18 S rRNA. The PCR products were electrophoresed through 1.5% agarose gel and ImageJ software was used to quantify the band intensity. Real-time PCR was performed using SyBR-green master mix (Applied Biosystems) on ABI prism 7500 sequence detection system. The relative levels of TLR expression was expressed as fold-change.

Primers for TLR2, TLR4 and 18 S were obtained from IDT and sequence are as follows: TLR 4 (S -5′-CCT-TGA-GAA-GGT-TGA-GAA-GTC-CCT-GC-3′(Tm = 60.9 °C); AS- 5′-GAA-GAT-GTG-CCT-CCC-CAG-AGG-3′(Tm = 59.7 °C)), TLR2 (S-5′-GGC-TGC-ACT-GGT-GTC-TGG-AG-3′ (Tm = 60.7 °C); AS-5′-CCC-AAT-GGG-AAT-CCT-GCT-CAC-TG-3′ (Tm = 60.0 °C)) and 18 S (S -5′-GTA-ACC-CGT-TGA-ACC-CCA-TT-3′(Tm = 55.3 °C); AS- 5′- CCA-TCC-AAT-CGG-TAG-TAG-CG-3′ (Tm = 55.1 °C)).

### FACS

Following treatment with morphine as described, cells were collected and fixed with IC Fixation Buffer (eBioscience; cat# 00-8222-49). The cells were subsequently stained with Anti-Mouse CD284 (TLR4) Alexa Flour 488 antibody (eBioscience; cat# 53-9041-80) for 30 min at 4 °C. Appropriate isotype control for anti-TLR was included. After washing with PBS, cells (100,000 events) were acquired by FACS Canto II cytometer and data was analyzed using Diva software (BD Bioscience).

### Phagocytosis of live bacteria and Cell Viability assay

J774 macrophage cells were pretreated with morphine (1 um) overnight. Cells were than incubated with either vehicle, LPS (50 ng/ml) or LTA (10 ug/ml) or 4 hours and then incubated with live GFP-tagged E. coli (1 × 10^3^) or live luciferase tagged S. aureus (1 × 10^3^) (bioluminescent strain 100) at a cell to bacteria ratio of 1:20. After 30 minutes of phagocytosis of live *bacteria,* J774 cells were washed four times in antibiotic (1% Penicillin/Streptomycin) containing PBS to eliminate proliferation and growth of non- internalized bacteria and washed in PBS and re-suspended in appropriate resistance antibiotic (Ampicllin for GFP *E. coli* and Kanamycin for *S. pneumonia).* Cell were than lysed and plated on bacterial growth plates (LB Ampicllin (100 ug/ml) for GFP *E. coli* and Kanamycin (50 ug/ml) blood agar for *S. pneumoniae*) and colonies were counted the next day.

To determine if incubation with the respective bacteria resulted in compromised cell viability, cell counts were evaluated using trypan blue exclusion method in the absence and presence of bacteria.

### Treatment with a p38 MAPK inhibitor SB203580

J774 cells were pretreated with 10 uM SB203580 (a p38 MAPK inhibitor) for 4 hours before overnight morphine (1 uM) incubation. SB203580 was maintained overnight, along with morphine. The next day cells were treated with either LPS or LTA and phagocytosis with gram (+) or gram (−) bacteria was evaluated for 30 minutes.

### Intracellular bacterial killing assay

J774 macrophage cells were plated in 6 well plates at 200,000 cells per well, in supplemented (antibiotic free) DMEM media. Following 16 hours of morphine (1 μM) treatment, cells were treated with LTA or LPS for 4 hours prior to addition of live GFP *E. coli* or *S. pneumoniae* (1 × 10^3^, in 1:20 cell:bacteria ratio). After 30 minutes of bacterial incubation cells were washed four times in antibiotic containing PBS to eliminate proliferation and growth of non-internalized bacteria. Cells were then incubated in antibiotic-free media for 24 hours to allow killing of internalized bacteria. LPS, LTA or morphine were added to the cells, where appropriate. Third day, following the overnight incubation, cells were washed, collected, lysed and plated on bacterial growth plates (LB Ampicllin (100 ug/ml) for GFP *E. coli* and Kanamycin (50 ug/ml) blood agar for *S. pneumoniae*) and colonies were counted the next day.

### Statistical analysis

The data are reported as the means ± SEMs of values of triplicate results. The data was analyzed using GraphPad Prism Software and a p value of 0.05 or less was considered significant for all the comparisons. The means of different treatments were compared using Student’s t test or ANOVA followed by Bonferroni’s correction if needed.

## Additional Information

**How to cite this article**: Jana, N. *et al.* Differential effects of gram-positive and gram-negative bacterial products on morphine induced inhibition of phagocytosis. *Sci. Rep.* **6**, 21094; doi: 10.1038/srep21094 (2016).

## Supplementary Material

Supplementary Information
