# Erratum: Differential effects of gram-positive and gram-negative bacterial products on morphine induced inhibition of phagocytosis

**DOI:** 10.1038/srep24011

**Published:** 2016-04-20

**Authors:** Jana Ninkovic, Vidhu Anand, Raini Dutta, Li Zhang, Anuj Saluja, Jingjing Meng, Lisa Koodie, Santanu Banerjee, Sabita Roy

Scientific Reports 6: Article number: 2109410.1038/srep21094; published online: 02192016; updated: 04202016.

The original version of this Article contained errors in the spelling of the authors Jana Ninkovic, Vidhu Anand, Raini Dutta, Lisa Koodie, Santanu Banerjee and Sabita Roy which were incorrectly given as Ninkovic Jana, Anand Vidhu, Dutta Raini, Koodie Lisa, Banerjee Santanu and Roy Sabita. These errors have now been corrected in the PDF and HTML versions of the Article.

