# Supplementary material for: Differential effects of gram-positive and gram-negative bacterial products on morphine induced inhibition of phagocytosis
Source: Sci Rep. 2016 Feb 19;6:21094. doi: 10.1038/srep21094 (PMC4759540; doi:10.1038/srep21094)
Supplement: Supplementary Information [file srep21094-s1.pdf]

Differential effects of gram-positive and gram-negative bacterial products on morphine induced inhibition of phagocytosis.

Ninkovic Jana+*, Anand Vidhu+*, Dutta Raini+, Li Zhang‡, Anuj Saluja+, Jingjing Meng+, Koodie Lisa+, Banerjee Santanu+ and Roy Sabita+, ‡

Supplemental Figure 1

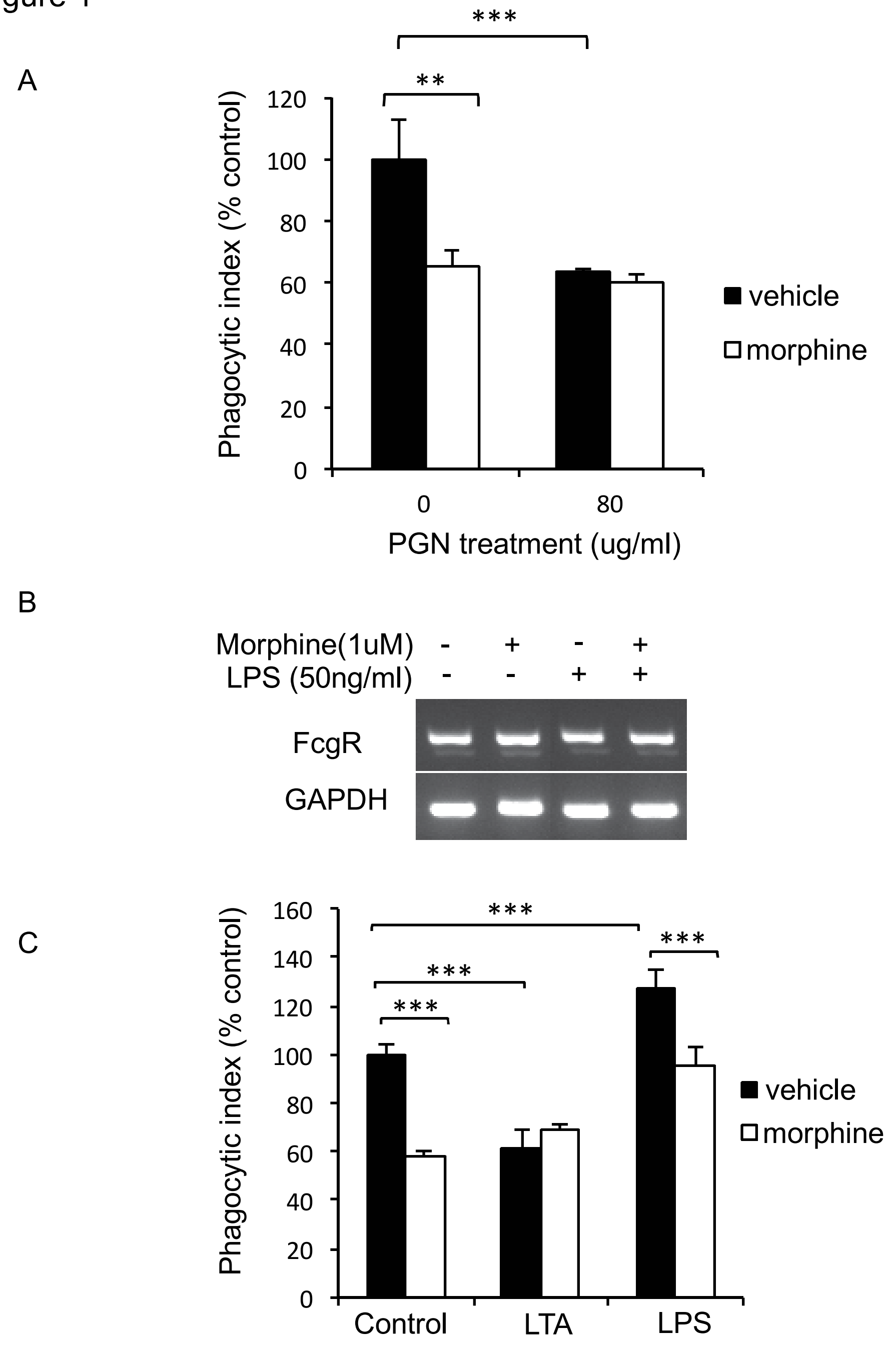

Supplemental Figure 1: Supplemental Figure 1: (A) Morphine inhibits phagocytosis of opsonized dextran beads. Treatment with Peptidoglycan from S. aureus (PGN- 80µg/ml) inhibits phagocytosis. (B) Short term LPS exposure (4hrs) not able to induce expression changes in FcγR1. (C) short-term morphine exposure (1µM for 4hr) in the presence of LPS (50ng/ml for 4 hours) was unable to induce morphine mediated increase in phagocytosis.

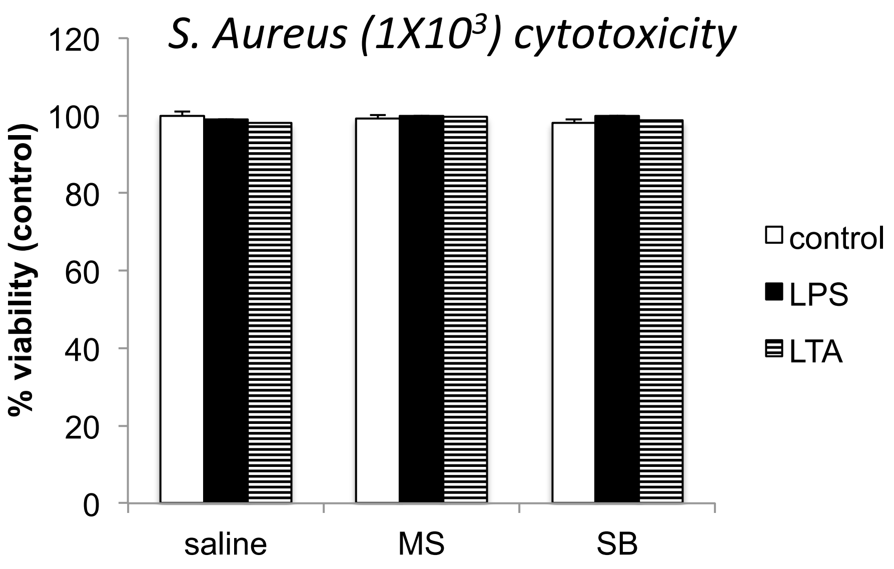

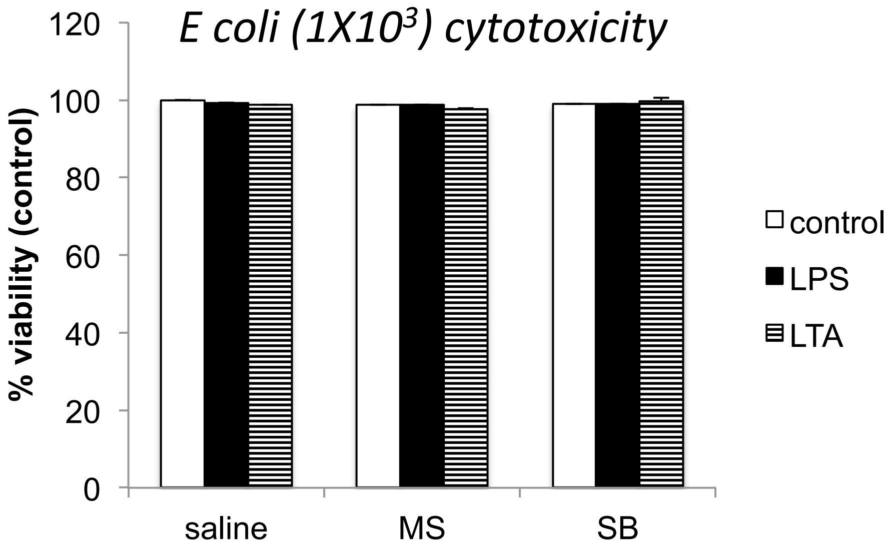

Supplemental Figure 2: Treatment of cells with live bacteria does not differentially alter cell viability cell Viability. J774 cells were pretreated with either Vehicle or SB for 4 hours and then treated with morphine (1uM) overnight. Cells were then exposed to live E. coli or S. aureus (bacteria to cell ratio 20:1) 30 minutes. Parallel wells were plated and not exposed to bacteria. Cells were then washed and viable cells determined using trypan blue staining and compared to wells that were not treated with bacteria (100%).
